# Quantification of Resting-State Ballistocardiogram Difference Between Clinical and Non-Clinical Populations for Ambient Monitoring of Heart Failure

**DOI:** 10.1109/JTEHM.2020.3029690

**Published:** 2020-10-08

**Authors:** Isaac Sungjae Chang, Susanna Mak, Narges Armanfard, Jennifer Boger, Sherry L. Grace, Amaya Arcelus, Caroline Chessex, Alex Mihailidis

**Affiliations:** 1 Institute of Biomaterials and Biomedical Engineering, University of Toronto 7938 ONM5S 3G9Canada; 2 Division of CardiologyDepartment of MedicineMount Sinai HospitalTorontoONM5G 1X5Canada; 3 Department of Electrical and Computer EngineeringMcGill University 5620 MontrealQCH3A 0G4Canada; 4 Department of Systems Design EngineeringUniversity of Waterloo 8430 WaterlooONN2L 3G1Canada; 5 Research Institute for AgingWaterlooONN2J 0E2Canada; 6 Faculty of HealthYork University 7991 TorontoONM3J IP3Canada; 7 Toronto Rehabilitation Institute, University Health Network 7989 TorontoONM5T 2S8Canada

**Keywords:** Ballistocardiogram, resting-state, heart failure, ambient monitoring

## Abstract

A ballistocardiogram (BCG) is a versatile bio-signal that enables ambient remote monitoring of heart failure (HF) patients in a home setting, achieved through embedded sensors in the surrounding environment. Numerous methods of analysis are available for extracting physiological information using the BCG; however, most have been developed based on non-clinical subjects. While the difference between clinical and non-clinical populations are expected, quantification of the difference may serve as a useful tool. In this work, the differences in resting-state BCGs of the two cohorts in a sitting posture were quantified. An instrumented chair was used to collect the BCG from 29 healthy adults and 26 NYHA HF class I and II patients while seated without any stress test for five minutes. Five 20-second epochs per subject were used to calculate the waveform fluctuation metric at rest (WFMR). The WFMR was obtained in two steps. The ensemble average of the segmented BCG heartbeats within an epoch were calculated first. Mean square errors (MSE) between different ensemble average pairs were then retrieved. The MSEs were averaged to produce the WFMR. The comparison showed that the clinical cohort had higher fluctuation than the non-clinical population and had at least 82.2% separation, suggesting that greater errors may result when existing algorithms were used. The WFMR acts as a bridge that may enable important features, including the addition of error margins in parameter estimation and ways to devise a calibration strategy when resting-state BCG is unstable.

## I. Introduction

In Canada, more than 660,000 people aged over 40 years had heart failure (HF) in 2013 [1], and 50,000 new cases of HF emerged yearly according to a 2016 report, costing more than $2.8 billion per year [2]. In the United States, HF cost over $11 billion and more than 1 million people were hospitalized due to HF in 2014 [3]. The high cost of HF is primarily attributable to the elevated rate of hospital readmission. Repeat visits, which happen in more than 20% of all HF patients, cause strain on both the patients and the healthcare system [2].

One of the strategies put in place to reduce the readmission rate is remote monitoring of the disease, namely self-management of the symptoms at home by the individuals with HF. The effectiveness of the method is compromised due to the low compliance rate [4]. Ambient monitoring is a methodology designed to achieve an autonomous and routine assessment in the background using embedded sensors, thereby promote patient compliance (i.e., symptoms are monitored in pervasive and unobtrusive manner) [5]. For example, an individual with HF can sit on a chair or sleep on a bed, where the embedded sensors in the objects will measure vital signs during the interaction, notifying the stakeholders when there is an impending adverse event.

A key bio-signal behind the mechanics of the ambient monitoring solution is a signal known as a ballistocardiogram (BCG). The BCG is defined as mechanical vibration generated by the heart contraction and the blood circulation; the phenomenon is also understood as the shift in the center of mass of the body due to the rapid movement of blood [6], [7]. The signal has shown substantial potential in improving the utility of remote monitoring owing to its non-contact nature and a direct reflection of the heart function [8].

One area of BCG research is using algorithmic solutions to extract physiological parameters. In the past studies, features of the BCG were correlated with and used to estimate the physiological parameters. These features were often retrieved in conjunction with other signals such as an electrocardiogram (ECG), photoplethysmogram (PPG), or impedance cardiogram (ICG). A structured review of these works revealed the research gaps that need to be addressed. Summaries of the studies are presented below for brevity. The correlated physiological parameters and the corresponding BCG features include heart rate (HR) using the J-waves of the BCG, pre-ejection rate (PEP) using RJ- and RI-intervals (i.e., the time interval between the R-wave of the ECG and the J- or I-wave of the BCG), cardiac output (CO) using the root mean square (RMS) power of the BCG, stroke volume (SV) using the combination of the I- and J-waves of the BCG with the ICG, systolic blood pressure (SBP) using the RJ-interval, and diastolic blood pressure (DBP) using the pulse transit time (i.e., time interval involving the I-wave of the BCG and PPG; PTT) [9]–[13], [21].

The above review elicited a crucial issue. BCGs in these works were measured from healthy adults with age below 65 (i.e., non-clinical population) using a weight scale (i.e., standing form). These analyses were done using an external stimulus to the physiology where the body experienced changes over time, which were correlated to the BCG features. Examples of the applied stimuli include the Valsalva maneuver, exercise, cold pressor test, and mental arithmetic [12]. The consensus of these studies was that the features remained stable at rest. The stimulated parameters returned to the baseline once the extent of the stimulus ended, and they remained relatively constant without any stress. This pattern, however, was not yet validated in a clinical population such as individuals with HF. As such, this assumption should be verified, starting with the simpler case where the BCG features remain stable in the absence of any stimulus thereafter moving onto conducting stress tests. If the BCG at rest were not stable, it might introduce errors when the above methods are used. In such a case, the degree of instability could be used as an indication of estimation confidence (i.e., a margin of error). Knowing the source of instability could be used to make a calibration method to compensate for the variability. These features become indispensable as the BCG research moves towards a clinical population with possibly unstable BCGS.

**FIGURE 1.**
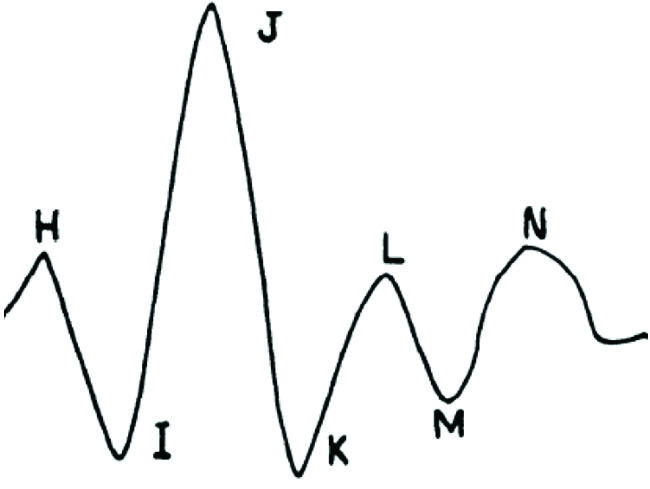
BCG morphology (duration and amplitude may vary based on external factors) [6].

A supplementary observation, in addition to the primary research gap above, was the dependency of the algorithms on specific points of one type of BCG waveform. Most of the methods relied on the conformity of the morphology to that of the longitudinal BCG measured when the individual was standing straight. Fig. 1 shows the morphology of the longitudinal BCG of a straight supine position that is also applicable to standing straight. Using the J- or I-wave of the BCG assumed that identifiable peaks were present in the signal. This assumption, however, could be violated if the morphology of the BCG took a different form. Note that a differing morphology does not refer to the changes manifest under a stimulus or any transient noise such as movement artifact; instead, it refers to permanent features of the morphology that can be seen at rest.

In the case of a significant difference, it may not be possible to identify the landmarks, in which the features cannot be extracted. On the contrary, a waveform-based feature that does not rely on a point could be generalized to different BCG morphologies. An example of a waveform-based feature is using the RMS power of the BCG. Regardless of the type, these features are affected by changes in the BCG. For instance, RMS power was primarily influenced by the amplitude change, and the features using the J-wave were often affected by the phase. Three factors were shown to influence the morphology of the BCG, which include: heart function, age, and posture, all of which are significant contributors when working with a clinical population (e.g., HF population) [14]–[19]. A detailed review of these factors is available in the Appendix.

It should be noted that a handful of studies are available that examined the BCG of HF patients. Notable works include differentiation of compensated state and the decompensated state, estimation of the SV, and ejection fraction [14], [16], [20]. There is currently no study that systematically examined the BCG of the clinical population at rest in comparison to the non-clinical cohort.

This work compared and quantified the difference between the resting-state BCGs of the non-clinical and clinical populations. Namely, the study involved healthy younger adults, a cohort used in the previous studies, and older adults with HF, a target population that will be using the BCG technology. The work examined whether the pattern observed in the non-clinical population at rest translated to the clinical population (i.e., stable waveform) through a novel BCG feature that quantified the collective fluctuation of the signal at rest. The feature then measured the degree of distinction between the populations if they were shown to be different. The new feature was also characterized via the comparison with different physiological parameters as a secondary analysis.

## II. Methods

In this work, a BCG feature was designed to compare and quantify the resting-state signal stability of the non-clinical population to that of the clinical cohort. The first part consisted of the study protocol, data acquisition, feature extraction, and comparison between the two populations.

In the second part, the developed feature was characterized by comparison with various physiological parameters.

### A. Comparison of BCG at Rest Between Non-Clinical and Clinical Populations

#### 1) Sample Populations and Experimental Setup

The study recruited older adults with the age of 65 years or older who have either the New York Heart Association (NYHA) class I or II HF. Healthy adults under 65 years of age who did not have any cardiovascular disease were recruited for the non-clinical population. HF patients were recruited from one of four ambulatory clinics: two heart function clinics and two cardiac rehabilitation programs at four academic hospitals in Toronto, Canada. The healthy participants were recruited through word of mouth and referrals. The study protocol was reviewed and approved by the University Health Network Research Ethics Board (UHN REB 12–038 and UHN REB 13-6901). The data were collected in the HomeLab at Toronto Rehab Institute in Toronto, Ontario, Canada.

Sitting posture was implemented to record the BCG that is closer to the actual deployment where alternative morphology state could be observed as compared to the standard morphology measured while standing still. The sitting posture was also selected for a practical purpose in this study. Limitation to stand still for one in five clinical participants for four to five minutes made much of the individual’s BCG unusable.

#### 2) Data Acquisition and Synchronization

Each participant, hereafter referred to as a subject, was asked to sit on an instrumented chair for one minute for the baseline restoration followed by five minutes without movement; data recorded during the stationary five-minute period was used for the analysis. No stimulation (e.g., exercise, cold pressor test, Valsalva maneuver) was given to the subjects for at least five minutes before the recording. Note that other parts of the protocol conducted but not related to this work were not considered herein.

**FIGURE 2.**
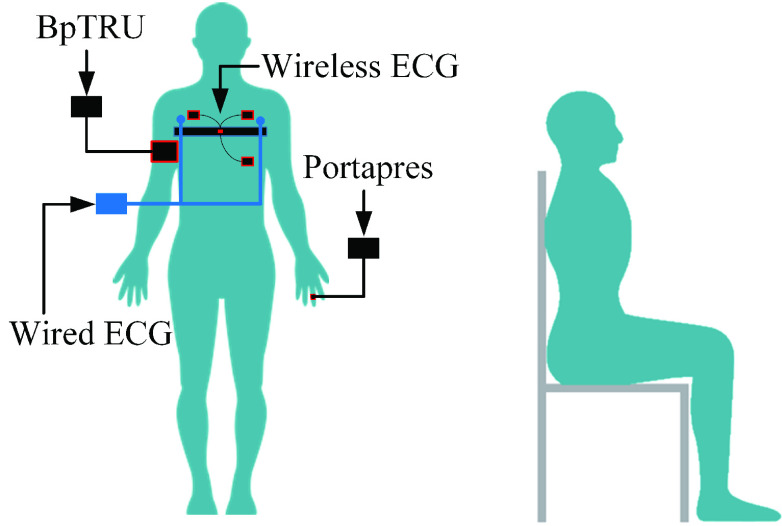
Gold standard equipment and the posture used to collect the data.

A 3-lead ECG was measured using the wireless Shimmer 3 ECG module (Shimmer, Ireland) to aid the BCG analysis. Also, beat-to-beat systolic and diastolic blood pressure (SBP; DBP) were measured using the Portapres (Finapres Medical Systems, the Netherlands) for the correlation analysis. Cuff-based BP was also measured every one-minute using the BpTRU (BpTRU Medical Devices, British Columbia, Canada). Fig. 2 illustrates the data collection setup. The wireless ECG was sampled at 1024Hz, and the rest of the signals were sampled at 1000Hz.

While the Portapres recorded beat-to-beat BP and showed changes over time, its measurements of absolute BP in mmHg were less accurate. As such, the beat-to-beat measurements of the Portapres were corrected to match the BP measured by the BpTRU. As the offset correction and synchronization processes were elaborated in previous work [21], the algorithms were briefly summarized here. The offset correction aimed to shift the entire sequence of BP samples to best match the BP measured by the BpTRU. About 1-minute of the beat-to-beat BPs from the Portapres before the BpTRU measurement were averaged. Then, the difference between the average and BP measured by BpTRU was calculated.

As the recording lasted five minutes, five of these differences were averaged and used as the offset correction constant to shift the entire beat-to-beat measurements of the Portapres. The SBP and DBP were corrected separately. Additional 2-lead wired ECG was collected for synchronization purposes. All signals except the wireless ECG were digitized by the National Instruments Data Acquisition Board (DAQ with a NI cDAQ-9174 chassis and NI 9215 analog input module) [9]. Matching the RR-intervals between the two ECGs synchronized them together and to the rest of the data. In five cases where the RR intervals were invariant (e.g., paced ECGs), a 3-axis accelerometer in the wireless ECG module was used to ascertain the synchronization by observing the transition from pre-recording (i.e., minor movement) to the stationary posture during the recording (i.e., no motion). Note that the wireless ECG was used as the primary ECG source as it was measured by a commercial gold-standard device.

#### 3) Hardware Specification of the Instrumented Chair

**FIGURE 3.**
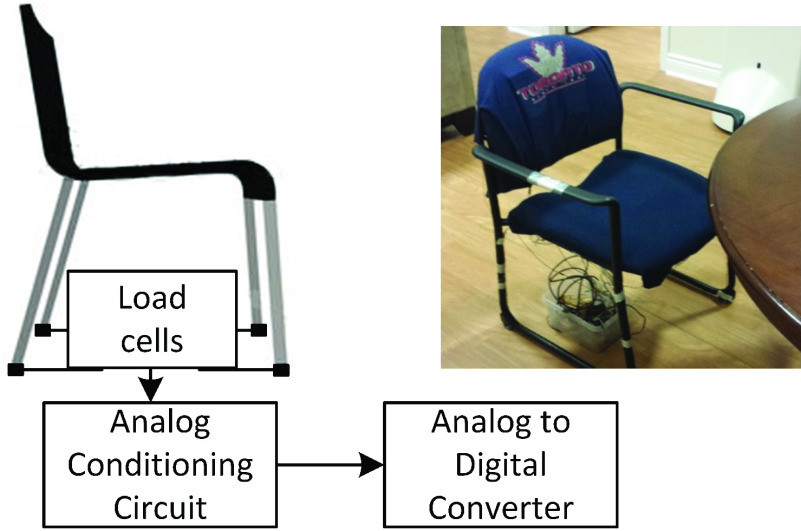
Instrumented chair prototype concept and the actual prototype.

The instrumented chair, hereafter referred to as the chair, followed the conventional setup of the weight scale, which is a widely accepted methodology for collecting the BCG [9], [10]–[13]. Four load cells were attached at the end of the chair’s legs, as shown in Fig. 3.

The load of the person seated on the chair was transferred through the four load cells. The chair and the feet supported the body weight, where the chair acted as the primary support. Chang *et al.* showed that while the BCG collected from the feet in a seated position was detectable, its amplitude was, on average, ten times smaller than that of the standing posture, indicating negligible force loss in detection due to the planted feet [22].

The four load cells formed a Wheatstone bridge and were conditioned using the circuit with about 100dB gain, presented in the previous work [9]. The circuit was used to acquire the BCG in a standing form in the prior work using the platform prototype. Thus, there was no difference between the acquisition process of the BCG for the standing form and sitting form used here. The processed signal was digitized by the DAQ at 1000 Hz.

#### 4) Pre-Processing

The wireless ECG was resampled using 1000Hz to match the other signals. A high-pass finite impulse response (FIR) filter filtered signal offsets and drifts with a cut-off frequency of 2Hz (i.e., Hamming window, n = 1000) [9]. Signals were also filtered by a low-pass filter. The BCG of all subjects and the ECG of the non-clinical population were filtered using 25Hz and 40Hz cut-off frequencies, respectively, as processed previously [9].

**FIGURE 4.**
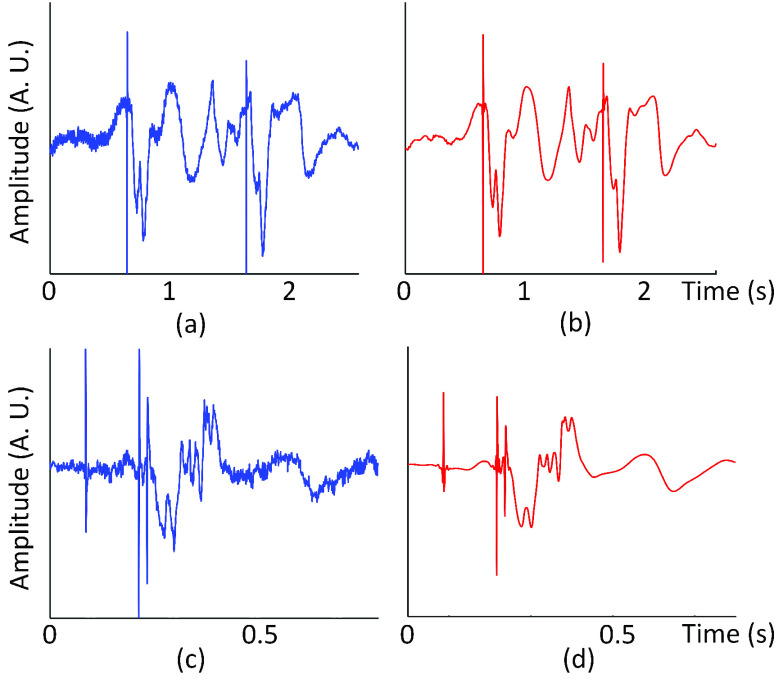
(a) (b) Lead II ECG before and after de-noising (subject C4) (c) (d) Lead III ECG before and after de-noising (subject C112).

The ECGs of the clinical population, however, were de-noised using discrete stationary wavelet transform due to their complex structure. Namely, the FIR low-pass filter or other digital filters were not used because they removed high-frequency components such as pacemaker pulses along with the white noise in the signal. An example of the ECG is shown in Fig. 4. Note that the process of de-noising the ECG of the clinical population involved numerous steps that stray from the primary analysis. As such, this section presents an abridged version, and the Appendix I includes the full explanation.

The ECG was decomposed into five levels using Daubechies 5 (db5) wavelet. A unique threshold value was used for each level to set the coefficients below the threshold to zero where the threshold values were determined based on the highest increase in the signal-to-noise ratio (SNR) while restricting any significant distortion of the waveform (i.e., less than 5% change).

#### 5) Waveform Fluctuation Metric at Rest

The feature used in this work was extracted once the signals were cleaned. The feature focused on its ability to compare the stability of the BCG at rest between the two populations. Namely, the feature quantified the degree of fluctuation in the waveform. While the existing features introduced above focused on particular aspects of the BCG (e.g., phase, amplitude), the feature developed here accounted for the collective effect of these changes; it is affected by the amplitude, phase, number of peaks, and an overall shift in shape. The feature provided a high-level pattern, and the following procedures were implemented with the goal in mind. The developed feature is hereafter called the waveform fluctuation metric at rest (WFMR).

##### a: Region Selection

**FIGURE 5.**
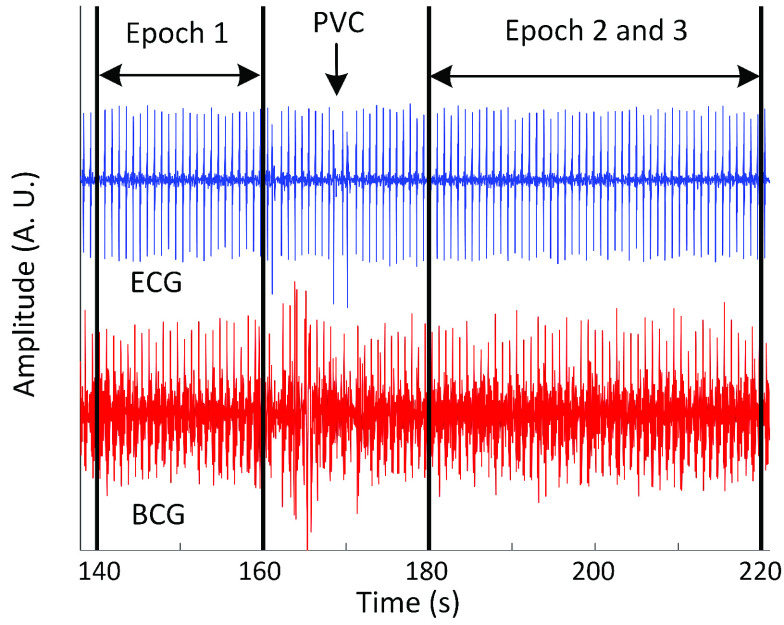
Selection of epochs for analysis (subject C41).

A specific duration of the signal had to be examined to calculate the WFMR. A few considerations were made to select the optimal length for the segment. Firstly, sections of the signal that represented the dominant type of heart contraction were selected. The ECG was used as the gold-standard measurement to identify the types as it provided a sharp contrast between different contractions. Some of the HF subjects had multiple types of heart contractions while the healthy population had only one type, which was an intrinsic heartbeat with sinus rhythm. These different types of contractions included paced heartbeats, premature ventricular contractions (PVC) in addition to the intrinsic heartbeat with sinus rhythm. Most of the HF subjects had a mixture of these three types; however, either an intrinsic or paced heartbeat was more dominant compared to the PVCs that occurred sporadically throughout the recording in some subjects. Note that the identification of different types of heartbeats was done in close supervision of a cardiologist to ascertain the reliability of the manual label. Each segment, hereafter called an epoch, was determined to be 20-second long to account for the effect of respiration on cardiac performance by including two or three respiratory cycles [23]. The size was also sufficiently small to provide flexibility to avoid non-dominant heartbeats in most cases. For example, if the dominant type was an intrinsic heartbeat with occasional PVCs, the epochs were placed so that there was no PVC within the range. If the primary type was a paced heartbeat with infrequent intrinsic heartbeats, the intervals were set so that only paced heartbeats were included. When mixing the types were unavoidable, non-dominant heartbeats were labelled and excluded from the analysis. An example of the epoch selection is shown in Fig. 5. Note that the clinical population has C in front of the subject number, and the non-clinical population has N in front of the number. Five-minute recording per subject was long enough for five epochs given the limited supply of homogeneous contractions in the clinical population.

##### b: Feature Calculation

Two steps were taken to calculate the WFMR. Firstly, the ensemble average, a widely used method for the BCG processing, was used to obtain noise-reduced BCG waveform, then mean square errors (MSE) between the ensemble averages in an epoch were calculated to obtain the WFMR.

The ensemble average effectively removed the noise that was not filtered by the FIR filter, namely, the low-frequency noise that existed within a similar frequency spectrum range as the BCG [24]. The R-waves of the ECG were first found by the simplified Pan and Tompkins method [25]. Each cardiac cycle was isolated by taking 700 samples (i.e., 700ms), beginning from the R-wave [24]. Note that each cropped cardiac cycle is hereafter referred to as the heartbeat. Given the window size of the ensemble average, $T_{EA}$, all heartbeats within the window were averaged. Equation (1) gives the mathematical description of the step.

\begin{equation*} f_{EA} \left ({t }\right)=\frac {1}{N_{EA}}\sum \limits _{i}^{N_{EA}} {f_{i} \left ({t }\right)} \quad {t=1\cdots n}\tag{1}\end{equation*}

$N_{EA}$ is the number of heartbeats that fit within the window size, $T_{EA}\cdot f_{i}(t)$ is a single cropped heartbeat with size, $n$, which is 700. $f_{EA}(t)$ is the ensemble average.

Different size for the ensemble average was evaluated where $T_{EA}$ was incremented from 2s to 15s by 1s to find the optimal range.

A moving window was used within an epoch to calculate multiple ensemble averages. The window shifted by 1s from the beginning until the window reached the epoch boundary. The moving window did not cross the boundary of epochs, even if two epochs were attached, as shown in Fig. 5. For example, using an 8-second window produced 12 ensemble averages for one epoch (i.e., 20-second long). Similarly, using a 5-second window resulted in 15 ensemble averages per epoch. The total number of ensemble averages per epoch was noted as $N_{Total}$. In rare cases, shifting the window by 1s resulted in the same set of heartbeats. In this case, the identical set was removed from the analysis, decrementing $N_{Total}$ by one. The calculation was repeated for all epochs.

The WFMR was calculated by taking the MSE ((2)) on every pair of the ensemble averages in an epoch. Higher MSE value resulted if there were a larger difference between the ensemble averages.

\begin{equation*} MSE=\sum \limits _{t=1}^{n} {\left ({{f_{EA} \left ({t }\right)-f_{EA}^{\prime } \left ({t }\right)} }\right)^{2}}\tag{2}\end{equation*}

The resulting MSEs in the epoch were then averaged according to (3). The equation was based on the matrix notation of all pairs of ensemble averages ($N_{Total} $ x $N_{Total}$) within the epoch and averaged the non-diagonal elements.

\begin{align*} WFMR_{T_{EA}} =\log \left ({{\frac {1}{N_{Total} \left ({{N_{Total} -1} }\right)}} }\right)\sum \limits _{j\ne i}^{N_{Total}} {\sum \limits _{i\ne j}^{N_{Total}} {MSE{}_{ij}}} \\\tag{3}\end{align*}

$WFMR_{T_{EA}}$ is the natural logarithm of the mean MSE for the epoch with the window size, $T_{EA}$. Natural logarithm was applied to spread the data points evenly as the values may form a concentrated cluster given a consistent waveform.

##### c: Optimal Window Size for Ensemble Average

$WFMR_{T_{EA}} $ was used to decide on the best window size for the ensemble average. Firstly, $WFMR_{T_{EA}} $ of all epochs of the subject were averaged. Then the subject averages were averaged across all subjects regardless of population. The total mean $WFMR_{T_{EA}} $ was plotted against $T_{EA}$, and the optimal window size was decided based on the graph. The chosen optimal window size was used throughout the rest of the analysis; thus, the notation, $T_{EA}$, was removed for brevity.

#### 6) Comparison of Non-Clinical and Clinical Populations

The WFMR was used to compare the resting-state BCG of the non-clinical and clinical populations. Five samples of the WFMR (i.e., five epochs) were available for each subject. These points were grouped according to the population and compared visually, statistically, and quantitatively using binary classification. The data of the two populations were plotted using a box plot. The distributions of the two populations were then compared using an unpaired t-test to assess if their means were statistically different. One-sided test with the significance level of 0.05 was used, and unequal variances between the populations were considered. The alternate hypothesis was that the distribution of the clinical population had higher mean than that of the non-clinical population (i.e., the clinical population had a higher fluctuation that that of the non-clinical population).

The binary classification task used naïve Bayes, logistic regression, and decision tree classifiers to separate the two cohorts for the quantitative result. Multiple classifiers generated different decision boundaries to identify any bias. Leave-one-subject-out (LOSO) partition was implemented, where the algorithm used a single subject’s data as the test set and the rest of the data as the training set. The number of maximum splits for the decision tree was adjusted from five to 30 to find the optimal parameter.

### B. Characterization of WFMR

It would be informative to understand which physiological parameters affected the WFMR so that the behavior could be traced back to the source. In this section, the relationship between the WFMR and other physiological parameters was investigated.

#### 1) Reference Feature Selection

The reference parameters were selected based on past studies and the merit of providing insights on the WFMR. The SBP, DBP, and RR-interval (i.e., heart rate) were used based on their relationship to the BCG shown in the previous works [9], [12]. In addition to the RR-interval, the ECG could provide information regarding the ventricular conduction system through the QRS interval and QTc interval. The QRS interval is defined as the conduction time of ventricular depolarization. The QTc interval is the time from the start of the ventricular contraction to the end of repolarization of the ventricle, corrected by the RR interval. It is the collective time of depolarization and repolarization [26]. These parameters were useful as the electrical conduction in HF patients could be uncoordinated, delayed, blocked, or have abnormal rhythm and rate that manifest as the inability of the heart to properly circulate the blood [27], [28]. A longer QRS interval is known to indicate a blocked or slowed conduction of electrical signals through the ventricles [26], [29]. A longer QTc interval indicates hindered repolarization of the heart muscle cells that limit a coordinated ventricular contraction [30].

#### 2) Reference Feature Extraction

While the BP-related parameters measured by the Portapres were ready to be used in the analysis, features from the ECG had to be extracted through the abridged steps explained below. Similar to the noise removal process, the full procedure is in the Appendix as this material diverges from the primary objective.

From the ECG, RR-interval was calculated by finding the time intervals between successive R-waves of the ECG. The ECG waveform was then segmented where its Q-, S-, and the end of the T-waves were labelled manually for the clinical population and using Hidden Markov Model (HMM) for the non-clinical population [31]. These labels were used to retrieve the QRS and QT intervals. The QT interval was further processed into two derivatives, the QT_BASE_ and QT_QRS_ intervals. The QT_BASE_ interval was set as an unadjusted version that was equivalent to the QT interval, and the QT_QRS_ interval was defined as the QT interval minus the portion of the QRS interval exceeding 120ms [32]. The QT_BASE_ interval included both the depolarization and repolarization, whereas the QT_QRS_ interval focused more on the repolarization. Both intervals were corrected based on the RR-interval using (4) [32], [33], which gave the final parameters used in the analysis, the QT$_{\mathrm {C, \textrm {} BASE}}$ and QT$_{\mathrm {C, QRS}}$ intervals.

\begin{equation*} QT_{C} =QT+0.154\left ({{1-RR} }\right)\tag{4}\end{equation*}

#### 3) Reference Feature Engineering

Similar analysis as the BCG ensemble average was applied to the reference parameters. The optimal window for the ensemble average was used to average the reference values within the window. Taking the QRS interval as an example, the intervals within the window of size, $T_{EA}$, were averaged. The process was repeated as the window moved by 1s shift. If the $T_{EA}$ were eight seconds, 12 averaged QRS intervals would result in one epoch. These 12 values were used to calculate the epoch mean and standard deviation (SD). The process was repeated for every epoch of each subject and all reference parameters. Natural logarithm was also applied subsequently.

#### 4) Reference Feature Comparison

The WFMR and the reference parameters were grouped according to the population and plotted using box plots (i.e., non-clinical population versus clinical population) using the labels N and C.

## III. Results

Twenty-six (26) NYHA Class I and II HF patients aged 65 and over and 29 healthy adults below 65 years of age were recruited for the clinical and non-clinical populations, respectively. The demographics of the populations are summarized in Table 1.

**TABLE 1:** Demographic Information of the Non-Clinical and Clinical Cohorts

	Non-clinical	Clinical
Sex	19 (65.5%)	19 (73.1%)
Age	29.1 ± 6.3 years	75.0 ± 6.8 years
NYHA Class (I)		7 (26.9%)
Body mass index	24.1 ± 6.3 kg/m^2^	28.7 ± 7.7 kg/m^2^

**FIGURE 6.**
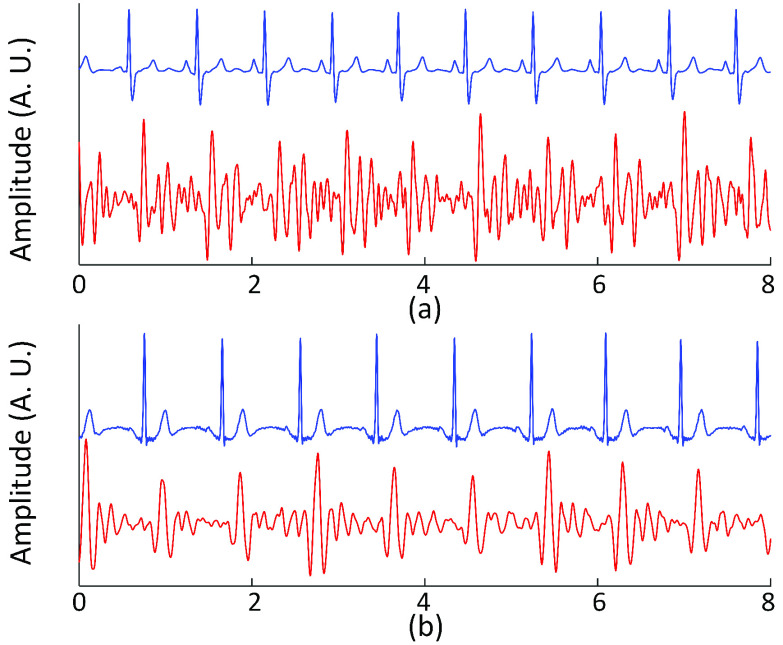
(a) ECG and BCG of a clinical subject in blue and red graphs respectively (Subject C51) (b) ECG and BCG of a non-clinical subject (subject N70).

**FIGURE 7.**
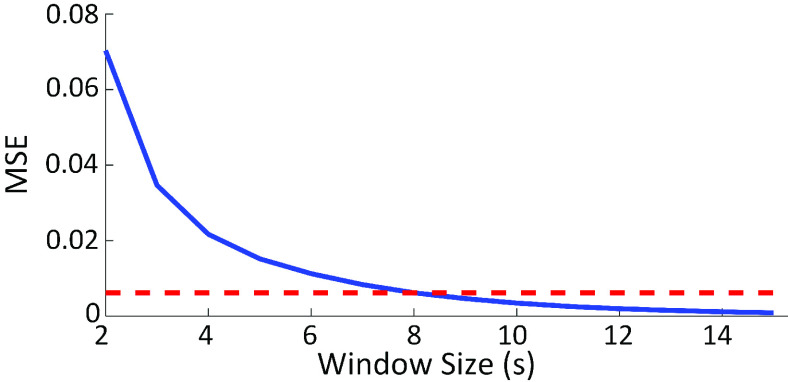
Optimal window size for ensemble average was decided as 8 seconds.

**FIGURE 8.**
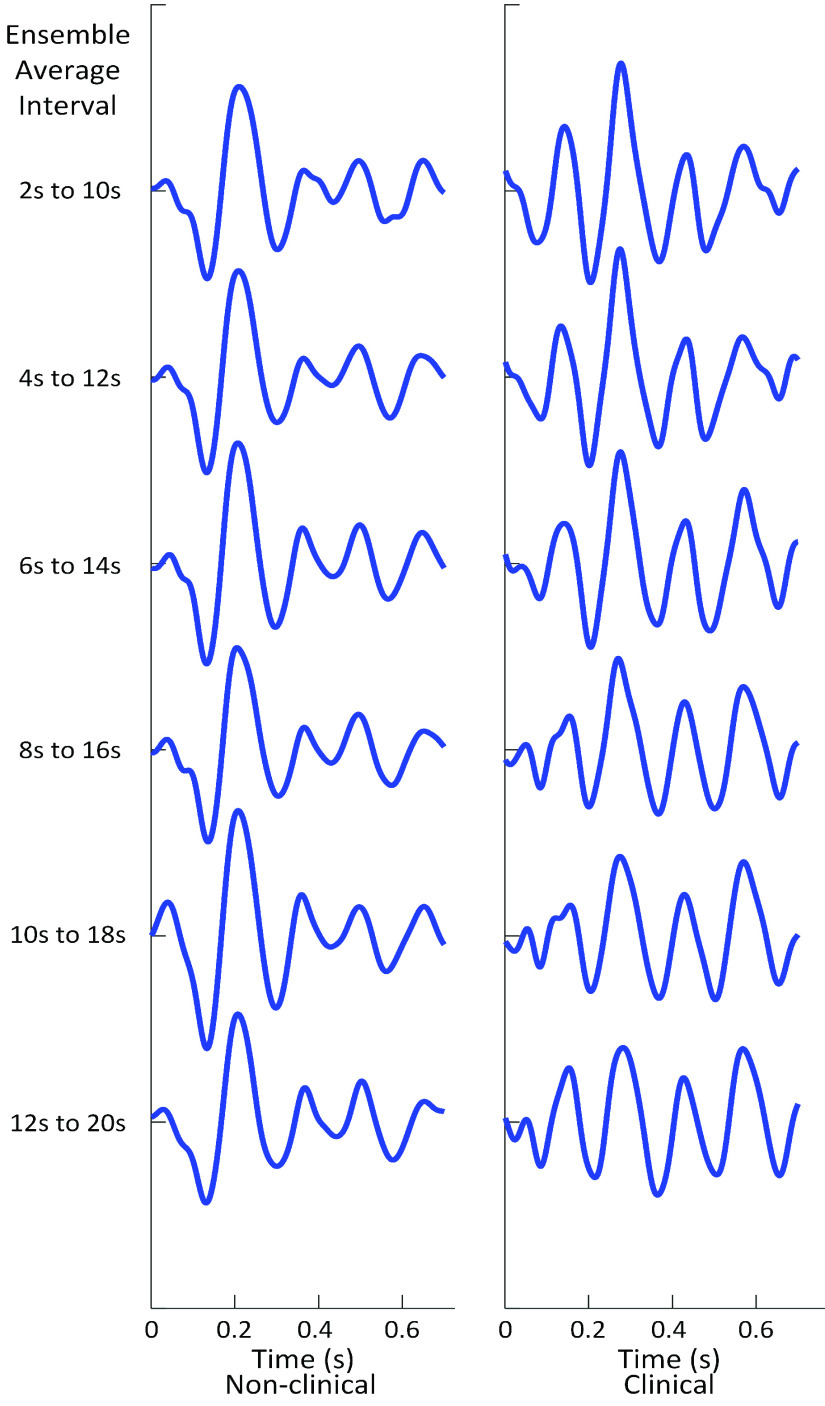
Ensemble averages of a non-clinical and clinical subject (subject N80 and C60) over one epoch. The change in waveform is evident in the clinical subject whereas minute change can be observed based on visual examination in a non-clinical subject.

**FIGURE 9.**
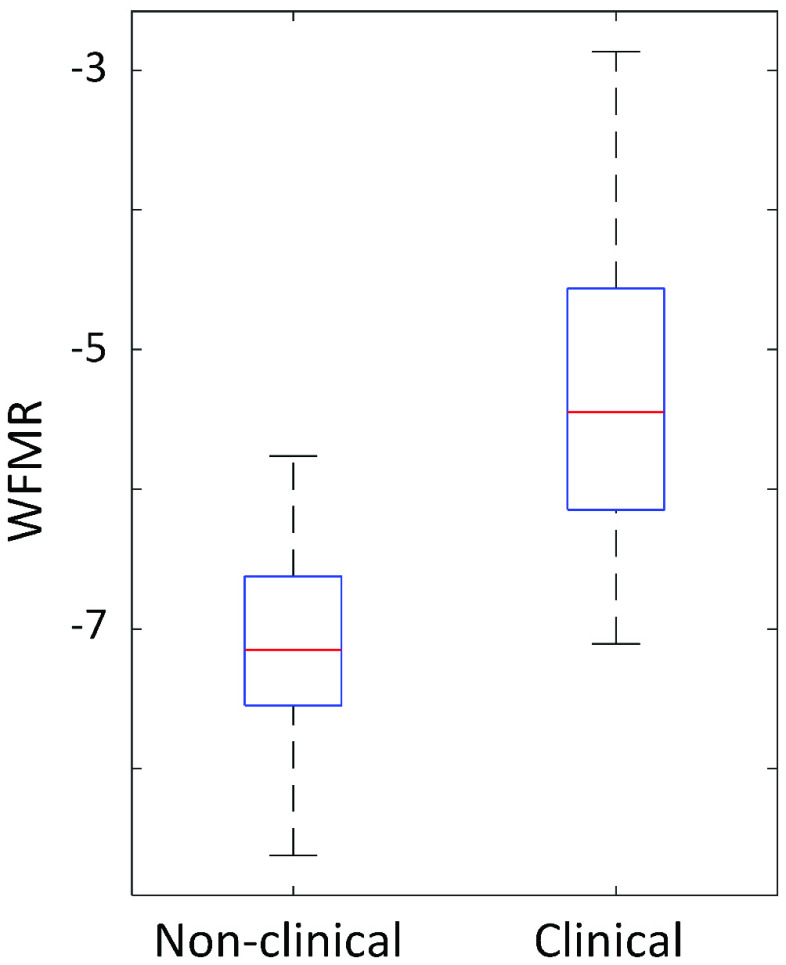
The WFMR shows higher variation of waveforms in the clinical population at rest.

BCG was successfully collected from all subjects, as shown in Fig. 6, and the WFMR was subsequently retrieved. The optimal window size was decided as 8 seconds, as shown in Fig. 7, since the reduction in mean MSE diminished marginally once the window size exceeded 8 seconds. Examples of the ensemble averages within an epoch for each subject of the two populations are shown in Fig. 8, which shows six ensemble averages over one epoch. According to the figure, the clinical cohort had a significantly higher dynamic than that of the non-clinical cohort. The box plot of the WFMR showed a more generalized pattern, where the clinical population had a much higher fluctuation in the waveform than that of the non-clinical population, as shown in Fig. 9. The t-test rejected the null hypothesis with the p-value of less than 0.001, indicating that the clinical population had a statistically higher mean WFMR than that of the non-clinical population.

Numerical analysis through binary classification resulted between 82.2% and 84.4% accuracy. The best number of maximum split values for the decision tree was seven. Note that different numbers of maximum splits had less effect on the accuracy where the result fluctuated only below 2-3% of the best results. The results are summarized in Table 2.

**TABLE 2:** Classification Results

Classifier	Naïve Bayes	Logistic regression	Decision tree
Accuracy	82.2%	84.4%	82.2%

In the second part of the analysis, the WFMR was compared to the reference parameters. In the process of extracting the parameters, a few subjects had to be removed due to technical difficulties. The end of T-waves in two clinical subjects (C41 and C53) were indeterminate based on the available resources. Therefore, the QT_C_ intervals of these two subjects were excluded from the analysis. The Portapres malfunctioned in four clinical subjects. As such, there were only 22 clinical subjects for the analyses involving SBP and DBP.

**FIGURE 10.**
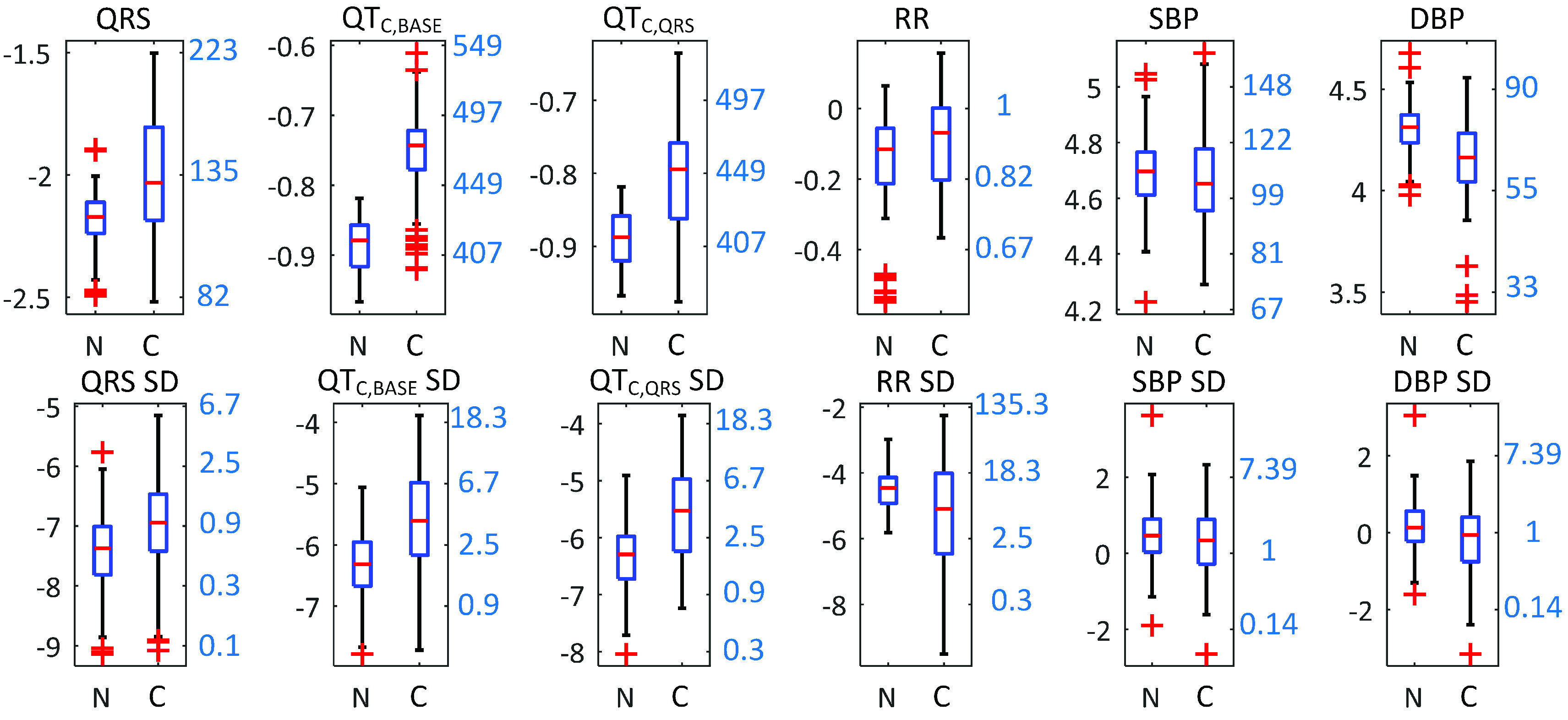
Box plots of other reference parameters comparing the non-clinical and clinical populations. Left y-axis shows the logarithmic scale and the right y-axis shows the converted numbers translated from the log scale. Units of translated y-axes are the following. QRS, QT$_{\mathrm {C,BASE}}$, QT$_{\mathrm {C,QRS}}$: ms; RR: s; SBP, DBP: mmHg.

A comparison of the distribution of the reference parameters between the non-clinical and clinical population showed that QT$_{\mathrm {C, BASE}}$, QT$_{\mathrm {C, \textrm {}QRS}}$, and QRS intervals showed similar patterns as the WFMR, as shown in Fig. 10.

## IV. Discussion

This work compared and quantified the distinction between the resting-state BCG of the clinical population and the non-clinical population using the WFMR. As shown in the results, the clinical population had a significantly more dynamic waveform over that of the non-clinical population at rest. The finding can have a crucial impact on the previously developed algorithms where the non-clinical population showed stability of the waveform at rest. Higher WFMR could imply that one or more physiological parameters related to the BCG (e.g., electrical conduction) were not stable at rest.

It is also possible that other unknown factors that were not accounted for created additional fluctuation in the BCG waveform. While the source of the variation will require exhaustive investigation, the secondary analysis provided some insight on the issue.

The methodology around the WFMR is not limited to the populations presented here. It is an effective tool that can be generalized to numerically measure the behavior of resting-state BCG in different cohorts.

**FIGURE 11.**
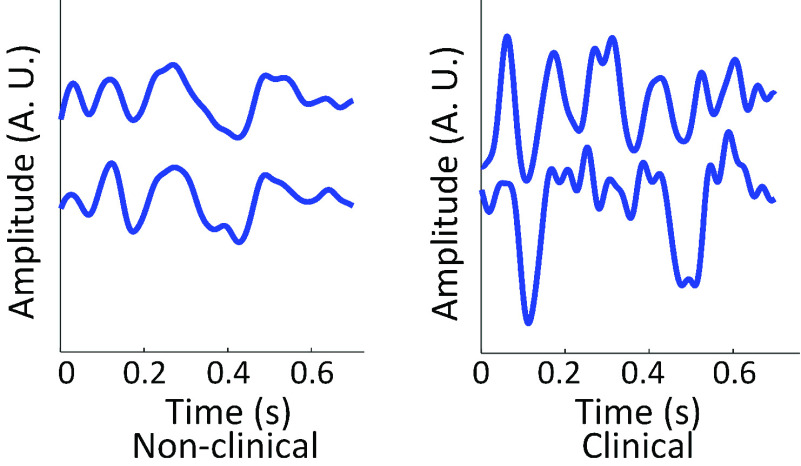
BCG ensemble averages that did not follow a typical morphology (subjects N73 and C7). The two ensemble averages shown in each graph are from different epochs.

Regarding the BCG acquisition, many of the signals were distinct from the widely used morphology of longitudinal BCG of a straight body, which agreed with the literature. The pattern was evident in both populations but with higher variation in the clinical population, as shown in Fig. 11. While the authors attempted to retrieve widely used features such as the RJ-interval, it was difficult or unattainable in some cases due to the difference present. This pattern was likely due to the non-standing posture, abnormal heart function of the clinical cohort, and age. When standing posture was evaluated for its usability in the clinical population, six out of 26 subjects could not stand still for a proper BCG measurement. Issues such as limited mobility, tremor, and frailty prevented the subjects from standing upright on a platform without any support, which rendered the collected BCG unusable.

Regarding the selection 8-second window, it should be noted that the exact size of the window matters less than the selection of the approximate region; other values in proximity (e.g., 7-second) would have a comparable effect. The use of similar window size has precedence in other works, including the authors’ previous works where the window size of 7-second for ensemble average was used. The effect of different window sizes has been elaborated in the article [21].

**FIGURE 12.**
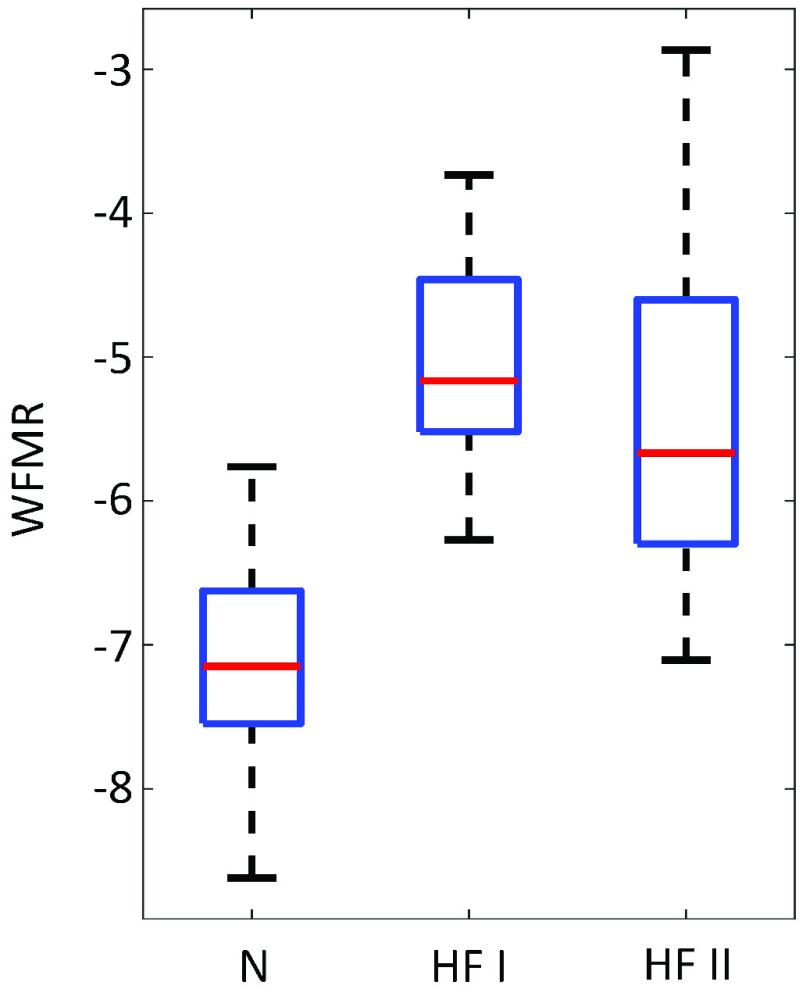
The WFMR of BCG for the non-clinical, HF I, and HF II. While the small sample size made the comparison inconclusive, there is no noticeable difference between class I and II.

In the classification task, there were four incorrectly classified subjects for the non-clinical population and six to seven for the clinical population depending on the classifier. The examination of the incorrectly classified subjects showed that the misclassified subjects in both classes had the WFMR lie in the overlapping region of the two distributions as expected. The data associated with these subjects indicated that the QRS interval, QT_C_ intervals, and the SD of these variables were all randomly spread around their respective distributions and did not show any pattern. Misclassified clinical subjects all had class II HF. Regarding any pattern within the clinical population, there was no noticeable difference in the WFMR between the class I and II subjects (Fig. 12). This observation, however, was based on limited data and should be further investigated, as there were only seven class I subjects. Interestingly, the ejection fraction (EF) retrieved from the clinical report form of these HF patients were quite broad.

Among the QT$_{\mathrm {C, BASE}}$, QT$_{\mathrm {C, QRS}}$, and QRS intervals that mimicked the behavior of the WFMR in the secondary analysis, QT$_{\mathrm {C, BASE}}$ showed the closest resemblance. Given that QT$_{\mathrm {C, QRS}}$ and QRS intervals focused on the repolarization and the depolarization, respectively and that QT$_{\mathrm {C, BASE}}$ was affected by both, the elevated WFMR was partially contributed by the elongated ventricular contraction due to the delay in the cardiac conduction. As supplementary evidence, linear regression between each of the three variables with the WFMR showed the correlation coefficients of 0.329, 0.523, and 0.353 for the QRS, QT$_{\mathrm {C, BASE}}$, and QT$_{\mathrm {C, QRS}}$ intervals, respectively. Note that this regression analysis was done by combining the two populations without controlling the independent variables, namely the heart function and age. As such, these results should be taken only as a secondary reference where the actual correlation between these parameters may differ. Interestingly, the electrical function exerted a certain role on the BCG while the signal’s origin was mechanical. The RR-interval and BP were not related to the WFMR, suggesting that the feature was not affected by rate- or vascular-related conditions (i.e., fluctuation at rest). This effect, however, requires further study as these parameters were shown to be influenced by the vascular system, and change may manifest during a stress test [34].

One of the future works is that the measurement of mechanical characteristics of the heart function (e.g., CO) in addition to the electrical characteristics should be taken. Namely, additional measurements such as echocardiogram or catheter measurements that provide the mechanical properties of the heart may reveal further information about the WFMR. With these measurements included, the future work should isolate the aspects of the electrical conduction and mechanical function to assess the degree of the influence each factor makes on the feature. As the study becomes more focused on the clinical aspect, relevant variables such as age and heart function should be controlled accordingly.

The concept of WFMR may be transferred to an application in two ways. The feature may be used to provide uncertainty in an estimation involving the BCG and suggest ways to calibrate the dynamic behavior of the resting-state BCG. The quantitative nature of the WFMR allows direct measurement of the fluctuation of BCG morphology at rest. As the fluctuation translates into the variability of the measured features (e.g., RJ-interval), the WFMR may be used to generate tolerance for an estimation. For example, if PEP is estimated using the BCG and ECG, the WFMR may be used to produce a signal quality index that can be used in calculating an error range or variance of the estimation. Based on this information, one can determine the reliability of the inferred parameter and decide to keep or discard the output. Secondly, the WFMR could enable ways to tune the dynamic behavior to make an algorithm based on the BCG more consistent. This implementation will, however, require further elucidation of the WFMR. Namely, understanding the source of the fluctuation may allow compensating for the influence. One way to address this issue is to categorize the WFMR into different types. Collective behavior analysis was sufficient to prove the potential of the WFMR.

However, branching the WFMR into different types may be necessary to attain a complete view of the resting-state behavior and understand the source of the fluctuation before undertaking any stress test. For example, changes in amplitude and phase can both affect the WFMR; grouping the signals according to these categories and performing a separate characterization for each may reveal ways to calibrate and re-use the previously developed algorithms during stress tests more effectively.

The presentation of the WFMR should be interpretable by the readers. The absolute value of the WFMR for each population separately present little value at the current stage. Instead, the feature should be understood in relative terms; comparison of the WFMR of one population (e.g., clinical) to a reference population (e.g., non-clinical) would provide the degree of variability in an intuitive manner. This was done using separation accuracy presented in a known metric of percentage in this work.

A few limitations were present in using the equipment. While the authors were highly confident of the features extracted, two HF subjects had to be removed from the QT_C_ analysis due to the limitation of using the 3-lead ECG. Using a 12-lead ECG in the future will eliminate this limitation. Lastly, the Portapres had malfunctioned in four clinical subjects. One notable source of the malfunction was a failure to detect the pulse at the extremity (i.e., a finger). This problem may surface again in the future as a specific portion of the HF patients have weaker circulation at the extremities, and the device such as the Portapres may not be able to detect the necessary pulse signal to make its measurements. It is advised that contingency forms of measurements be prepared in case the problem resurfaces.

Additional directions for future research include expanding the current analysis to examine non-time domain characteristics of the WFMR, such as frequency components, transformation coefficients. This work included only mild to moderate HF patients. It is expected that further differentiation will occur when more severe HF (i.e., NYHA class III and IV) patients are included. Later study should assess the capability of the system to assist HF patients self-manage their condition. This includes the efficacy to unobtrusively detect adverse events related to HF in a home environment. The effectiveness of the WFMR may be evaluated in cohorts with different health conditions to further validate the efficacy of the feature to generalize. Lastly, the source of morphology fluctuation, as well as the morphology alteration, may be identified with the help of mathematical models, where the matching the waveforms by tuning parameters may narrow down the source of observations made in this work [35], [36].

## V. Conclusion

This work investigated the resting-state BCG of the non-clinical and clinical population using the WFMR. The analysis showed that the clinical population had a statistically higher fluctuation of the waveform at rest with a minimum 82.2% separation, which may adversely affect the performance of the existing algorithms in estimating the physiological parameters. To achieve ambient monitoring of older adults with HF successfully, the WFMR may be used to compute a margin of errors via signal quality index when a conventional algorithm is used. As well, the behavior of the WFMR should be elucidated further to identify the source of the fluctuation for calibration prior to conducting stress tests on the clinical population.

## References

[1] Heart Disease in Canada: Highlights From the Canadian Chronic Disease Surveillance System, Public Health Agency Canada, Ottawa, ON, Canada, 2017.

[2] 2016 Report on the Health Of Canadians: The Burden of Heart Failure, Heart Stroke Found. Canada, Ottawa, ON, Canada, 2016.

[3] Jackson S. L., Tong X., King R. J., Loustalot F., Hong Y., and Ritchey M. D., “National burden of heart failure events in the united states, 2006 to 2014,” Circulat., Heart Failure, vol. 11, no. 12, Dec. 2018, Art. no. e004873. 10.1161/CIRCHEARTFAILURE.117.004873 PMC6424109 30562099

[4] Holden R. J., Schubert C. C., and Mickelson R. S., “The patient work system: An analysis of self-care performance barriers among elderly heart failure patients and their informal caregivers,” Appl. Ergonom., vol. 47, pp. 133–150, Mar. 2015. 10.1016/j.apergo.2014.09.009 PMC4258227 25479983

[5] Boger J., Mihailidis A., and Hoey J., Zero Effort Technologies: Considerations, Challenges, and Use in Health, Wellness, and Rehabilitation, 2nd ed. San Rafael, CA, USA: Morgan & Claypool, 2018.

[6] Starr I., Rawson A. J., Schroeder H. A., and Joseph N. R., “Studies on the estimation of cardiac ouptut in man, and of abnormalities in cardiac function, from the heart’s recoil and the blood’s impacts; the ballistocardiogram,” Amer. J. Physiol.–Legacy Content, vol. 127, no. 1, pp. 1–28, 1939.

[7] Starr I. and Noordergraaf A., Ballistocardiography in Cardiovascular Research: Physical Aspects of the Circulation in Health and Disease. Philadelphia, PA, USA: Lippincott, 1967.

[8] Inan O. T., “Ballistocardiography and seismocardiography: A review of recent advances,” IEEE J. Biomed. Health Inform., vol. 19, no. 4, pp. 1414–1427, Jul. 2015. 25312966 10.1109/JBHI.2014.2361732

[9] Chang I. S. J., Javaid A. Q., Boger J., Arcelus A., and Mihailidis A., “Design and evaluation of an instrumented floor tile for measuring older adults’ cardiac function at home,” Gerontechnology, vol. 17, no. 2, pp. 77–89, Aug. 2018.

[10] Inan O. T., Etemadi M., Paloma A., Giovangrandi L., and Kovacs G. T. A., “Non-invasive cardiac output trending during exercise recovery on a bathroom-scale-based ballistocardiograph,” Physiol. Meas., vol. 30, no. 3, pp. 261–274, Mar. 2009. 19202234 10.1088/0967-3334/30/3/003

[11] Etemadi M., Inan O. T., Giovangrandi L., and Kovacs G. T. A., “Rapid assessment of cardiac contractility on a home bathroom scale,” IEEE Trans. Inf. Technol. Biomed., vol. 15, no. 6, pp. 864–869, Nov. 2011. 21843998 10.1109/TITB.2011.2161998

[12] Martin S. L.-O., “Weighing scale-based pulse transit time is a superior marker of blood pressure than conventional pulse arrival time,” Sci. Rep., vol. 6, no. 1, p. 39273, Dec. 2016. 27976741 10.1038/srep39273 PMC5157040

[13] Ashouri H., Orlandic L., and Inan O. T., “Unobtrusive estimation of cardiac contractility and stroke volume changes using ballistocardiogram measurements on a high bandwidth force plate,” Sensors, vol. 16, no. 6, p. 787, 5 2016, doi: 10.3390/s16060787. PMC4934213 27240380

[14] Conn N. J., Schwarz K. Q., and Borkholder D. A., “In-home cardiovascular monitoring system for heart failure: Comparative study,” JMIR mHealth uHealth, vol. 7, no. 1, Jan. 2019, Art. no. e12419. 10.2196/12419 PMC6356186 30664492

[15] Starr I. and Noordergraaf A., Ballistocardiography in Cardiovascular Research Physical Aspects of the Circulation in Health and Disease. Amsterdam, Netherlands: North-Holland, 1967.

[16] Zhang X., Zhang L., Wang K., Yu C., Zhu T., and Tang J., “A rapid approach to assess cardiac contractility by ballistocardiogram and electrocardiogram,” Biomed. Eng./Biomedizinische Technik, vol. 63, no. 2, pp. 113–122, Mar. 2018. 10.1515/bmt-2015-0204 27824610

[17] Goedhard W. J., “Ballistocardiography: Past, present and future,” Bibliotheca Cardiol., vol. 37, pp. 27–45, Jan. 1979. 508258

[18] Giovangrandi L., Inan O. T., Wiard R. M., Etemadi M., and Kovacs G. T. A., “Ballistocardiography—A method worth revisiting,” in Proc. Annu. Int. Conf. IEEE Eng. Med. Biol. Soc., Aug. 2011, pp. 4279–4282. 10.1109/IEMBS.2011.6091062 PMC4274997 22255285

[19] Javaid A. Q., Wiens A. D., Fesmire N. F., Weitnauer M. A., and Inan O. T., “Quantifying and reducing posture-dependent distortion in ballistocardiogram measurements,” IEEE J. Biomed. Health Inform., vol. 19, no. 5, pp. 1549–1556, Sep. 2015. 26058064 10.1109/JBHI.2015.2441876 PMC4560978

[20] Aydemir V. B., “Classification of decompensated heart failure from clinical and home ballistocardiography,” IEEE Trans. Biomed. Eng., vol. 67, no. 5, pp. 1303–1313, 5 2020. 31425011 10.1109/TBME.2019.2935619 PMC7271768

[21] Chang I. S., Armanfard N., Javaid A. Q., Boger J., and Mihailidis A., “Unobtrusive detection of simulated orthostatic hypotension and supine hypertension using ballistocardiogram and electrocardiogram of healthy adults,” IEEE J. Transl. Eng. Health Med., vol. 6, pp. 1–13, 2018. 10.1109/JTEHM.2018.2864738 PMC6193524 30345183

[22] Chang S. I., “Passive physiological monitoring via ambient sensors embedded in a home environment,” Inst. Biomater. Biomed. Eng., Univ. Toronto, Toronto, ON, Canada, 2019.

[23] Wright S. P., “The pulmonary artery wedge pressure response to sustained exercise is time-variant in healthy adults,” Heart, vol. 102, no. 6, pp. 438–443, Mar. 2016. 26762239 10.1136/heartjnl-2015-308592

[24] Etemadi M., Inan O. T., Wiard R. M., Kovacs G. T. A., and Giovangrandi L., “Non-invasive assessment of cardiac contractility on a weighing scale,” in Proc. Annu. Int. Conf. IEEE Eng. Med. Biol. Soc., Sep. 2009, pp. 6773–6776. 10.1109/IEMBS.2009.5332508 19963690

[25] Pan J. and Tompkins W. J., “A real-time QRS detection algorithm,” IEEE Trans. Biomed. Eng., vol. BME-32, no. 3, pp. 230–236, Mar. 1985. 10.1109/TBME.1985.325532 3997178

[26] Fauci A. S., Harrison’s Principles of Internal Medicine. New York, NY, USA: McGraw-Hill Education, 2008.

[27] (5 7, 2018). Heart Failure Workup. [Online]. Available: https://emedicine.medscape.com/article/163062-workup#c12

[28] Nicholson C., Heart Failure: A Clinical Nursing Handbook. Hoboken, NJ, USA: Wiley, 2007, p. 31.

[29] Surawicz B., “AHA/ACCF/HRS recommendations for the standardization and interpretation of the electrocardiogram: Part III: Intraventricular conduction disturbances a scientific statement from the American heart association electrocardiography and arrhythmias committee, council on clinical cardiology; the American college of cardiology foundation; and the heart rhythm society endorsed by the international society for computerized electrocardiology,” J. Amer. College Cardiol., vol. 53, no. 11, p. 976, 2009. 10.1016/j.jacc.2008.12.013 19281930

[30] Rautaharju P. M., Surawicz B., and Gettes L. S., “AHA/ACCF/HRS recommendations for the standardization and interpretation of the electrocardiogram: Part IV: The ST segment, T and U waves, and the QT interval: A scientific statement from the American heart association electrocardiography and arrhythmias committee, council on clinical cardiology; the American college of cardiology foundation; and the heart rhythm society. endorsed by the international society for computerized electrocardiology,” J. Amer. College Cardiol., vol. 53, no. 11, pp. 982–991, Mar. 2009. 10.1016/j.jacc.2008.12.014 19281931

[31] Roman K., Egor I., Alexander K., and Dmitry P., “CardIO library for deep research of heart signals,” Zenodo, Jan. 2018, doi: 10.5281/zenodo.1156086.

[32] Sagie A., Larson M. G., Goldberg R. J., Bengtson J. R., and Levy D., “An improved method for adjusting the QT interval for heart rate (the Framingham heart study),” Am. J. Cardiol., vol. 70, no. 7, p. 797, 1992. 1519533 10.1016/0002-9149(92)90562-d

[33] Vandenberk B., “Which QT correction formulae to use for QT monitoring?” J. Amer. Heart Assoc., vol. 5, no. 6, Jun. 2016, Art. no. e003264, doi: 10.1161/JAHA.116.003264. PMC4937268 27317349

[34] Yousefian P., “Physiological association between limb ballistocardiogram and arterial blood pressure waveforms: A mathematical model-based analysis,” Sci. Rep., vol. 9, no. 1, pp. 019–5146, Mar. 2019. 10.1038/s41598-019-41537-y PMC6435670 30914687

[35] Guidoboni G., “Cardiovascular function and ballistocardiogram: A relationship interpreted via mathematical modeling,” IEEE Trans. Biomed. Eng., vol. 66, no. 10, pp. 2906–2917, Oct. 2019. 30735985 10.1109/TBME.2019.2897952 PMC6752973

[36] Kim C.-S., “Ballistocardiogram: Mechanism and potential for unobtrusive cardiovascular health monitoring,” Sci. Rep., vol. 6, no. 1, p. 31297, Aug. 2016. 27503664 10.1038/srep31297 PMC4977514

[37] Wusk G. and Gabler H., “Non-invasive detection of respiration and heart rate with a vehicle seat sensor,” Sensors, vol. 18, no. 5, p. 1463, 5 2018, doi: 10.3390/s18051463. PMC5982527 29738456

[38] Islam M. K., Tangim G., Ahammad T., and Khondokar M. R. H., “Study and analysis of ECG signal using MATLAB & labview as effective tools,” Int. J. Comput. Electr. Eng., vol. 4, no. 3, p. 404, 2012.

[39] (2019). Analyzing Harmonic Distortion. [Online]. Available: https://www.mathworks.com/help/signal/examples/analyzing-harmonic-distortion.html

[40] Panicker G. K., “Drug-induced QT prolongation when QT interval is measured in each of the 12 ECG leads in men and women in a thorough QT study,” J. Electrocardiol., vol. 47, no. 2, pp. 155–157, 2014. 24388488 10.1016/j.jelectrocard.2013.11.004

[41] Goldberger A. L., “PhysioBank, physiotoolkit, and physionet: Components of a new research resource for complex physiologic signals,” Circulation, vol. 101, no. 23, pp. E215–E220, Jun. 2000. 10851218 10.1161/01.cir.101.23.e215

[42] (Sep. 8, 2013). Conduction. [Online]. Available: https://en.ecgpedia.org/index.php?title=Conduction

